# Flow cytometry FRET reveals post-translational modifications drive Protein Phosphatase-5 conformational changes in mammalian cells

**DOI:** 10.1016/j.cstres.2024.10.002

**Published:** 2024-10-10

**Authors:** Rebecca A. Sager, Sarah J. Backe, Jennifer Heritz, Mark R. Woodford, Dimitra Bourboulia, Mehdi Mollapour

**Affiliations:** 1 Department of Urology, SUNY Upstate Medical University, NY 13210, USA; 2 Upstate Cancer Center, SUNY Upstate Medical University, NY 13210, USA; 3 Department of Biochemistry and Molecular Biology, SUNY Upstate Medical University, NY 13210, USA

**Keywords:** Serine/threonine Protein Phosphatase-5, Flow cytometry, Fluorescence resonance energy transfer, SUMOylation, Phosphorylation, Post-translational modification

## Abstract

The serine/threonine Protein Phosphatase-5 (PP5) plays an essential role in regulating hormone and stress-induced signaling networks as well as extrinsic apoptotic pathways in cells. Unlike other Protein Phosphatases, PP5 possesses both regulatory and catalytic domains, and its function is further modulated through post-translational modifications (PTMs). PP5 contains a tetratricopeptide repeat (TPR) domain, which usually inhibits its phosphatase activity by blocking the active site (closed conformation). Certain activators bind to the PP5–TPR domain, alleviating this inhibition and allowing the catalytic domain to adopt an active (open) conformation. While this mechanism has been proposed based on structural and biophysical studies, PP5 conformational changes and activity have yet to be observed in cells. Here, we designed and developed a flow cytometry-based fluorescence resonance energy transfer (FC-FRET) method, enabling real-time observation of PP5 autoinhibition and activation within live mammalian cells. By quantifying FRET efficiency using sensitized emission, we established a standardized and adaptable data acquisition workflow. Our findings revealed that, in a cellular context, PP5 exists in multiple conformational states, none of which alone fully predicts its activity. Additionally, we have demonstrated that PTMs such as phosphorylation and SUMOylation impact PP5 conformational changes, representing a significant advancement in our understanding of its regulatory mechanisms.

## Highlights

• Use of a novel flow cytometry fluorescence resonance energy transfer method for PP5.
• FC-FRET revealed multiple conformational states of PP5 in real time within live cells.
• PP5 primarily is in a closed state but not completely inactive in living cells.
• Phosphorylation and SUMOylation drive conformational changes and regulate PP5 in cells.

## Introduction

Protein Phosphatase-5 (PP5) is a serine/threonine protein phosphatase that regulates a number of cellular functions, including stress and hormone responses, proliferation, apoptosis, and DNA repair.^1^, ^2^ Many of these regulatory functions of PP5 are a result of its role as a co-chaperone of the molecular chaperone heat shock protein 90 (Hsp90). Since the catalytic and regulatory domains of PP5 are encoded on a single polypeptide, PP5 adopts an autoinhibited conformation.^3^, ^4^, ^5^ Its extreme C-terminal αJ helix interacts with the tetratricopeptide repeat (TPR) domain in the N-terminus and blocks substrate access to the catalytic site, resulting in low basal activity.^5^ Interaction between the TPR domain and Hsp90 or polyunsaturated fatty acids activates PP5, classically thought by autoinhibition release.^6^, ^7^, ^8^, ^9^ We have previously shown that post-translational modification of PP5 also affects its activity and substrate release. Casein kinase 1δ-mediated phosphorylation of PP5-T362 leads to hyperactivity and supports kidney cancer survival.^10^, ^11^ Additionally, we have recently demonstrated that PP5 SUMOylation causes substrate release both *in vitro* and *in vivo*.^12^ Interestingly, phosphorylation of PP5 appears to be a prerequisite for its SUMOylation.^12^

Several structural studies of PP5 *in vitro* have identified motifs essential for its function.^13^, ^14^, ^15^ Catalytic activity is dependent on two metal ions interacting with several key residues of PP5.^4^ Mutation of these key residues, including PP5-H304, renders PP5 catalytically dead.^4^ Further studies investigating the structure of PP5 in complex with substrates contributed significantly to our understanding of PP5 substrate-contacting residues.^13^ The crystal structure of PP5 was solved in complex with a peptide fragment of the co-chaperone substrate Cdc37, and additional structures of PP5 in complex with Hsp90, Cdc37, and kinase clients BRAF and CRAF were recently solved by the Pearl and Agard groups.^13^, ^14^, ^15^ Both the autoinhibited and open conformations of PP5 were observed, revealing that the open conformation switches between the two TPR-binding sites of the Hsp90 dimer to allow access to various sites within the substrate client.^14^ Additionally, the structure from the Agard group suggested that client kinase release from Hsp90 must precede dephosphorylation of Cdc37 by PP5, as opposed to previously thought.^15^, ^16^

Classically, PP5 is considered to exist in a “closed” and autoinhibited conformation, and binding to activators such as Hsp90 leads to adoption of an “open” conformation that allows substrate access to the binding pocket.^5^, ^6^, ^17^, ^18^, ^19^ However, the conformational changes sampled by PP5 in a cellular context and the impact of PTMs on these conformational states remain elusive. Here, we developed an *in vivo* fluorescence resonance energy transfer (FRET) system to monitor the population dynamics of PP5 in live cells.

## Results

### Validation of PP5 FRET in cells

**Fig. 1:**
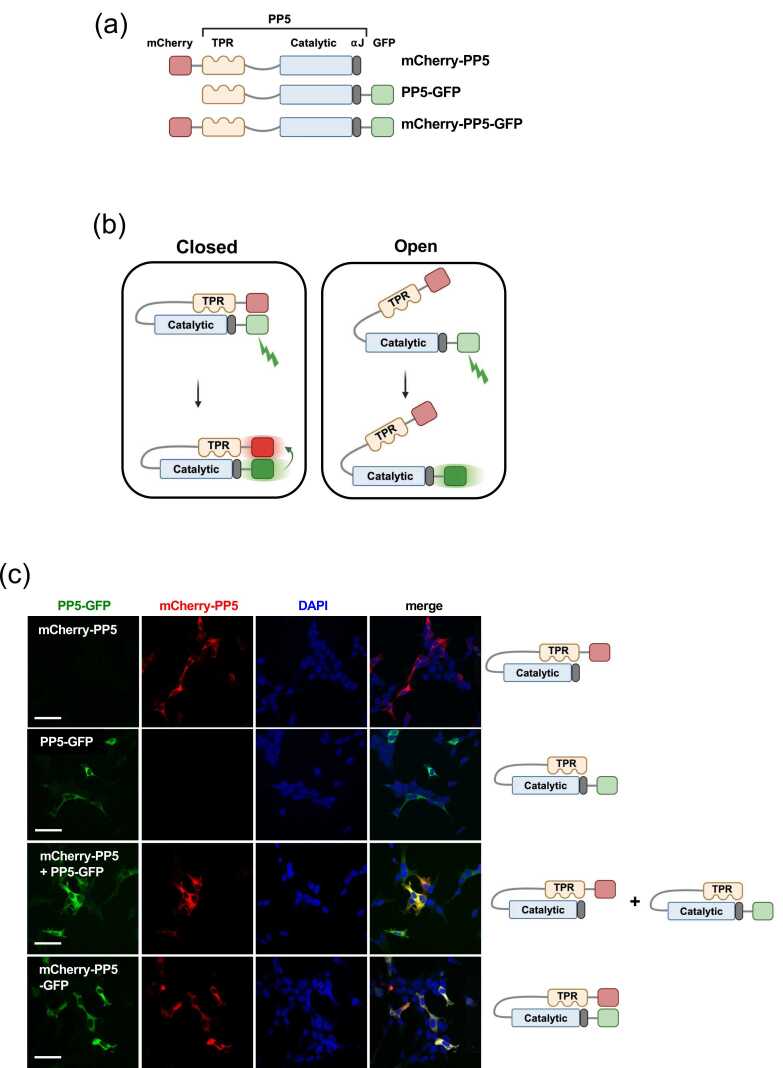
Validation of PP5 FRET in live cells. (a) Schematic representation of the domains of PP5, including the tetratricopeptide repeats (TPR) and extreme C-terminal αJ helix as well as fusion of GFP and mCherry proteins. (b) Schematic representation of FRET and emission from mCherry (in red) following excitation of GFP (in green) only when PP5 is in a “closed conformation.” Excitation of GFP with PP5 in an “open conformation” does not result in FRET. (c) HEK293 cells were transiently transfected with mCherry–PP5, PP5–GFP, or mCherry–PP5–GFP. DAPI was used for nuclear staining. Fixed cells were analyzed by fluorescence microscopy. The scale bar represents 50 µm. Abbreviations used: DAPI, 4′,6-diamidino-2-phenylindole; FRET, fluorescence resonance energy transfer; PP5, Protein Phosphatase-5.

To monitor the population dynamics of PP5 in live cells, we developed an *in vivo* FRET system where mCherry was attached to the N-terminus of PP5 and green fluorescent protein (GFP) to its C-terminus, both together and individually (Figure 1(a)). We anticipated that, when PP5 adopted a “closed” conformation, the two fluorescent proteins would come into close proximity, and excitation of GFP at 488 nm would allow for energy transfer to mCherry and detection of mCherry emission at 610 nm (Figure 1(b)). When PP5 adopted an “open” conformation, however, FRET would be lost (Figure 1(b)). We next characterized wild-type PP5 by transiently expressing PP5 N-terminally tagged with the fluorescent proteins mCherry and C-terminally tagged with eGFP in HEK293 cells (Figure 1(c)). The mCherry–PP5–eGFP fusion protein had similar activity as the untagged PP5 *in vitro* (data not shown). Additionally, mCherry–PP5–eGFP intracellular localization was not affected (Figure 1(c)).

### Visualization of FRET in live cells

**Fig. 2:**
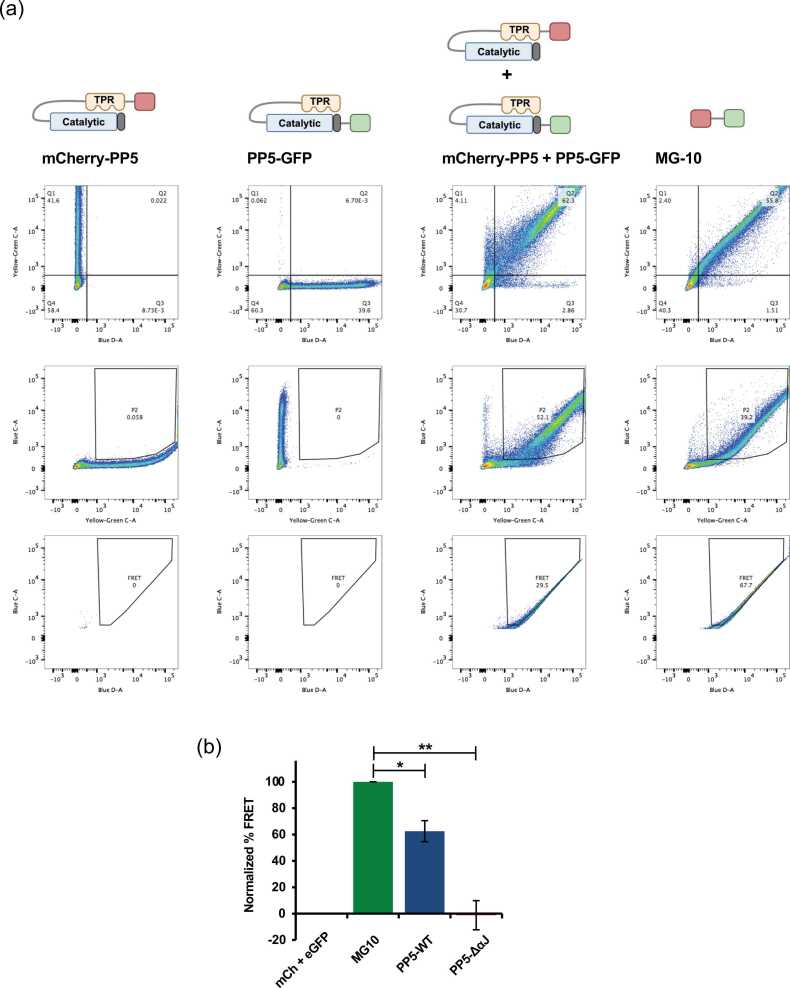
Visualization of FRET in live cells. (a) Flow cytometry data demonstrating a gating strategy for calculating FRET positive percentage. HEK293 cells were transfected with the indicated constructs. Gating for yellow green-C (mCherry) *versus* blue-D (GFP) in the top row using mCherry–PP5 and PP5–GFP constructs to isolate the population positive for both fluorophores and to exclude single positive populations. Population P2 isolated on blue-C (FRET) *versus* yellow green-C (mCherry) in order to correct for background signal in the FRET channel from the presence of mCherry alone (middle row). In the bottom row, the FRET population gated within population P2 by excluding the background of collisional FRET using the mCherry–PP5 + PP5–GFP sample and FRET positive population seen in the MG-10 positive control. Gating strategy adapted from.^20^, ^21^ (b) Normalized FRET determined *via* flow cytometry following transient expression of PP5 WT and nonautoinhibited PP5–ΔαJ. Co-expression of mCherry–PP5 and PP5–GFP used as a negative control to correct for collisional FRET. MG-10 used as a positive control for maximal FRET efficiency. Error bars represent the standard deviation of three independent experiments. A Student’s t-test was performed to assess statistical significance compared to MG-10 positive control (*P < 0.05; **P < 0.01). Abbreviations used: FRET, fluorescence resonance energy transfer; PP5, Protein Phosphatase-5; mCherry, mCh.

We utilized a flow cytometry-based approach for measuring FRET (FC-FRET) adapted from that described by others.^20^, ^21^, ^22^ We used co-expression of mCherry–PP5 and PP5–GFP as a negative control for collisional FRET and MG-10, which contained mCherry and GFP separated by a short linker optimized for FRET efficiency, as a positive FRET control.^23^ Gating of the results as shown in Figure 2(a) allowed the determination of the relative FRET signal. These were used as positive and negative controls for normalization to determine the “percent FRET” of cells expressing mCherry–PP5–GFP–WT or mCherry–PP5–GFP–ΔαJ (Figure 2(b)). We detected the FRET of the mCherry–PP5–GFP construct, suggesting the WT protein has a mixed state of open and closed conformations (Figure 2(b)). Examination of the active PP5–ΔαJ, however, showed a loss of FRET signal, as expected, because this mutant can no longer adopt a “closed” and autoinhibited conformation (Figure 2(b)). This confirmed that our assay reliably detected the loss of FRET as a result of PP5 conformational change.

### PP5 conformation dynamics by FC-FRET in live cells

**Fig. 3:**
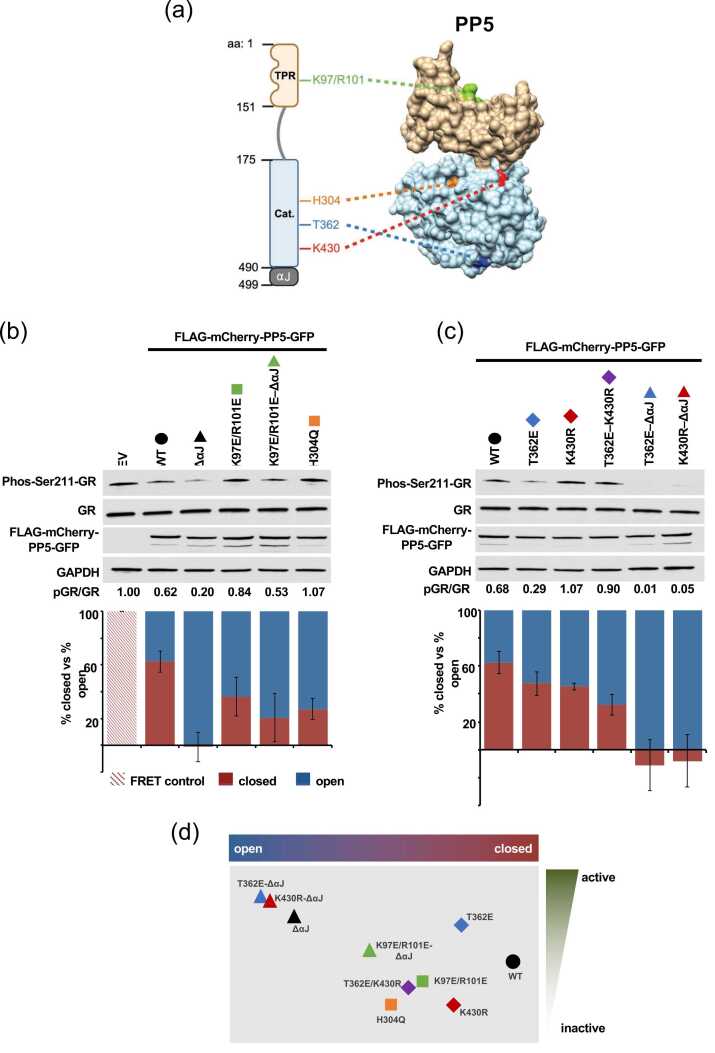
PP5 conformation dynamics by FC-FRET in live cells. (a) PP5 K97/R101 binding sites in the TPR-domain, catalytic site H304, phosphorylation T362 residue, and SUMOylation K430 site schematic representation (left) and modeled on “closed” PP5 structure (PDB: 7zr5) using UCSF Chimera (right). (b) Relative proportion of PP5 WT and mutants in open and closed conformation as determined by FC-FRET. Co-expression of mCherry–PP5 and PP5–GFP used as a negative control to background subtract to correct for collisional FRET. MG-10 used as a positive control to set maximal FRET efficiency at 100%. Western blot demonstrates the relative expression level of mutants and PP5 activity by GR–S211 phosphorylation. Error bars represent the standard deviation of three independent experiments. (c) Relative proportion of PP5–WT and PTM mutants in open and closed conformation as determined by FC-FRET. Co-expression of mCherry–PP5 and PP5–GFP used as a negative control to background subtract to correct for collisional FRET. MG-10 used as a positive control to set maximal FRET efficiency at 100%. Western blot demonstrates the relative expression level of mutants and PP5 activity by GR–S211 phosphorylation. Error bars represent the standard deviation of three independent experiments. (d) Relative proportion of PP5 mutants in open *versus* closed conformation as determined by FC-FRET in (b) and (c) *versus* PP5 activity. Activity assessed by densitometry of GR–S211 phosphorylation by Western blot corrected for total GR (b and c). Abbreviations used: FC-FRET, flow cytometry-based fluorescence resonance energy transfer; GR, glucocorticoid receptor; PP5, Protein Phosphatase-5.

We next used *PP5* functional mutants to further examine the relationship between PP5 activity and its conformational change. We used PP5–K97E/R101E mutant, which prevents substrate binding to the TPR-domain, catalytically dead PP5–H304Q mutant, PP5–ΔαJ mutant representing the open conformation, phospho-mimetic PP5–T362E and non-SUMOylatable PP5–K430R mutants, alone or in combination (Figure 3(a)). We used immunoblotting to ensure equal expression of our PP5 constructs in HEK293 cells (Figure 3(b)). Glucocorticoid receptor (GR) is a *bona fide* substrate of PP5. Specifically, PP5 dephosphorylates phos–Ser211–GR.^24^ Our data demonstrated that the TPR-domain binding site mutant (PP5–K97E/R101E), as well as the catalytically dead PP5-H304Q mutant, demonstrated more “open” conformations than PP5–WT *via* FC-FRET analysis, but these are both notably inactive towards substrate dephosphorylation (Figure 3(b)). Additionally, the PP5–K97E/R101E–ΔαJ mutant has an open conformation, similarly to PP5–ΔαJ; however, it is less active than PP5–ΔαJ with regards to substrate dephosphorylation (Figure 3(b)). Our data challenge the current dogma and strongly suggest that there is not necessarily a correlation between PP5 conformation and activity within cells.

We next assessed the impact of PTMs on PP5 conformation. Our data revealed that the phospho-mimetic PP5–T362E, non-SUMOylatable PP5–K430R, and double T362E–K430R mutants all displayed slightly more open conformations by FC-FRET than PP5–WT (Figure 3(c)). As mentioned earlier, these mutants all displayed different phosphatase activities. The phospho-mimetic PP5–T362E is hyperactive compared to PP5–WT while both the K430R and T362E–K430R mutants are entirely inactive in cells (Figure 3(c)). Additionally, deletion of the αJ helix in the context of these PTM site mutants opened the conformation and yielded an active phosphatase having bypassed the need for PTM (Figure 3(c)). Collectively, our FC-FRET analysis suggested that, in a cellular context, WT-PP5 primarily is in a closed state but not completely inactive. Additionally, our data suggested that structural conformation is not singularly predictive of PP5 activity and that post-translational modification of PP5 alters its conformation (Figure 3(d)).

## Discussion

Classically, PP5 is thought to have a low basal activity due to the adoption of a “closed” and autoinhibited conformation that restricts substrate access to its active site.^3^, ^4^, ^5^ Binding of activators, such as Hsp90 or Hsp70, could then release this autoinhibition and lead to an “open” and active conformation.^6^, ^7^, ^8^, ^9^, ^25^ Recent structural work, however, revealed that PP5 is in both a closed and autoinhibited state as well as a more open conformation in complex with Hsp90, Cdc37 co-chaperone, and a kinase substrate/client.^14^

We previously demonstrated that PTMs, including T362 phosphorylation and K430 SUMOylation, play a major role in the modulation of PP5 activity within a cellular context.^1^, ^10^, ^11^, ^12^ Specifically, our prior works have shown that phosphorylation of T362 is a prerequisite to SUMOylation of PP5–K430.^10^, ^12^ This ordered series of PTMs was found to be essential for PP5 substrate dephosphorylation and release. The challenge remained in understanding how PTMs affect PP5 conformation and detecting these changes in cells.

We designed and developed a flow cytometry-based FRET assay to analyze PP5 conformations in live mammalian cells, addressing the limitations of previous *in vitro* studies. Utilizing a series of PP5 point mutants, we found that the PTM of PP5 appears to alter its conformation, but there was no consistent correlation between activity of PP5 and its conformation. This suggests that PP5 samples multiple conformational states as it interacts with and dephosphorylates its substrates. However, its activation and regulation are more dynamic and complex than a structural release of autoinhibition. We are not often able to visualize chaperone machinery within live cells. Further development and adaptation of techniques such as FC-FRET are essential to our understanding of the differences in dynamics of these complex systems in a cellular context.

## Conclusion

In conclusion, we have successfully developed a novel FC-FRET method, which allowed us to observe conformational states of PP5 in real time within live mammalian cells. Our findings demonstrated that, in a cellular context, PP5 primarily exists in a closed conformation, though it is not completely inactive. Additionally, we showed that PTMs, such as phosphorylation and SUMOylation, significantly influence the conformational dynamics and regulatory function of PP5. These insights enhance our understanding of complex regulatory mechanisms in cellular environments and provide a valuable tool for future studies.

## Materials and methods

### Cell lines

Cultured human embryonic kidney (HEK293) cells were grown in Dulbecco’s Modified Eagle Medium (Sigma-Aldrich) supplemented with 10% fetal bovine serum (Sigma-Aldrich). HEK293 cells were acquired from the American Type Culture Collection. Cells were maintained in a CellQ incubator (Panasonic Healthcare) at 37°C in an atmosphere containing 5% CO_2_.

### Plasmids

For mammalian expression, pcDNA3–PP5–FLAG was created previously.^10^ MG-10, pCMV–mCherry, and pEGFP–N1 were obtained from Dr Barry Knox.^23^ The mCherry–PP5–GFP constructs were subcloned using the primers listed in Table S1. Point mutations were made using site-directed mutagenesis using primers as previously published^10^, ^12^ (see Table S1) and confirmed by DNA sequencing.

### Cell transfection

Cultured HEK293 cells were split and then transfected the following day when about 40% confluent with plasmid DNA using Mirus TransIT-2020 according to the manufacturer’s protocol. Cells were extracted or collected for analysis the following day.

### Protein extraction, immunoprecipitation, and immunoblotting

Protein extraction from mammalian cells was carried out using methods previously described.^12^, ^26^, ^27^ The lysis buffer contained (20 mM Tris-HCl (pH 7.4), 100 mM NaCl, 1 mM MgCl_2_, 0.1% NP40, protease inhibitor cocktail (Roche), and PhosSTOP (Roche)) with the addition of 20 mM *N*-ethylmaleimide. Protein samples were diluted with 5× Laemmli buffer and were separated by Sodium dodecyl sulfate-polyacrylamide gel electrophoresis (SDS-PAGE), and transferred to nitrocellulose membranes. Proteins from cell lysate were detected by immunoblotting with antibodies recognizing Rabbit anti-GR ((D6H2L) Cell Signaling Technology), Cat# 12041; RRID:AB_2631286, Rabbit anti-phospho-GR S211 Cell Signaling Technology, Cat# 4161; RRID:AB_2155797, Rabbit anti-FLAG Thermo Scientific Cat# PA1-984B; RRID:AB_347227, and Mouse anti-GAPDH ((1D4) Enzo Life Sciences) Cat# ADI-CSA-335; RRID:AB_10617247. Secondary antibodies raised against mouse anti-mouse secondary (Cell Signaling Technology) Cat# 7076; RRID:AB_330924, and anti-rabbit secondary (Cell Signaling Technology) Cat# 7074; RRID:AB_2099233. Western blot densitometry analysis was performed using ImageJ.

### Fluorescence microscopy

HEK293 cells were plated overnight on glass coverslips (#1) and then transiently transfected with mCherry–PP5, PP5–GFP, or mCherry–PP5–GFP overnight. Cells were fixed in 4% paraformaldehyde for 20 min at room temperature, washed 3× with fresh PBS, and then permeabilized with 0.1% Triton X-100 at room temperature for 4 min followed by 3 washes with fresh phosphate buffered saline (PBS). Coverslips were then mounted onto glass slides using ProLong® Gold antifade mounting media with 4′,6-diamidino-2-phenylindole (ThermoFisher Scientific). Images were obtained using a Zeiss LSM780 confocal microscope.

### FC-FRET

Cultured cells were transfected with fluorescent constructs, as described above. After overnight transfection, cells were trypsinized, washed with PBS, and resuspended in PBS containing 2 mM ethylenediaminetetraacetic acid (EDTA) and 0.5% fetal bovine serum. Flow cytometry-based FRET measurements were performed using a BD LSRII Cell Analyzer equipped with standard 488 and 561 nm lasers. To measure GFP fluorescence intensity, the cells were excited with the 488 nm laser, and fluorescence was collected with a 530/30 band pass filter. For FRET intensity, cells were excited with the 488 nm laser, and fluorescence was collected with a 610/20 band pass filter. To measure mCherry intensity, cells were excited with the 561 nm laser, and fluorescence was collected through the 610/20 band pass filter. A minimum of 10,000 fluorescence-positive cells were collected from each sample in each experiment. Analysis of flow cytometry data was performed using FlowJo software. Gating and determination of relative FRET proportions were adapted from Trumper *et al*^20^ and Banning *et al*^21^ and as seen in Figure 2(a). Gating of the flow cytometry data was performed to isolate only that population expressing both mCherry and PP5 and to exclude any false positive signal in the FRET channel from only excitation of mCherry (P2 in Figure 2(a)). Plotting FRET *versus* GFP then allowed us to draw a FRET gate to distinguish between the collisional FRET in the mCherry–PP5 and PP5–GFP co-transfected sample and the FRET seen from the MG-10 positive control. The mCherry–PP5 + PP5–GFP positive proportion was background subtracted from the other samples. MG-10 was then set at 100%, and a relative % FRET proportion was determined for the mCherry–PP5–GFP samples. FRET positive cells were assumed to represent the closed conformation of PP5, and FRET negative to represent the open conformation.

### Quantification and statistical analysis

All statistics were performed using GraphPad Prism version 9.2.0 for macOS (GraphPad Software, La Jolla, CA, USA, www.graphpad.com). Error bars represent the standard deviation for three independent experiments. Significance is denoted by asterisks in each figure: *P < 0.05; **P < 0.01.

### Protein structure modeling

UCSF Chimera v1.14 was used to model the PP5 protein structure (PDB: 7zr5) deposited by Oberoi *et al*.^14^

## Funding and support

This work was supported by the National Institute of General Medical Sciences of the National Institutes of Health under Award Number R35GM139584 (M.M.). The content is solely the responsibility of the authors and does not necessarily represent the official views of the National Institutes of Health. This work was also supported with funds from the SUNY Upstate Medical University and Upstate Foundation.

## Author contributions

**Dimitra Bourboulia**, **Jennifer Heritz**, **Rebecca A. Sager**: Data curation, Formal analysis, Writing – original draft. **Mark R. Woodford:** Data curation, Formal analysis. **Sarah J. Backe:** Data curation, Formal analysis, Methodology, Writing – original draft. **Mehdi Mollapour:** Conceptualization, Data curation, Formal analysis, Funding acquisition, Investigation, Methodology, Project administration, Resources, Supervision, Visualization, Writing – original & final draft, Writing – review & editing.

## Declarations of interest

The authors declare the following financial interests/personal relationships which may be considered as potential competing interests: Professor Mehdi Mollapour reports financial support was provided by the National Institute of General Medical Sciences. The authors MM, MRW, and DB are members of the CSAC editorial board. However, they were not involved and were unable to access any reviewing process and decision making of their manuscript. If there are other authors, they declare that they have no known competing financial interests or personal relationships that could have appeared to influence the work reported in this paper.

## Data Availability

No data were used for the research described in the article.
