# Supplementary material for: Flow cytometry FRET reveals post-translational modifications drive Protein Phosphatase-5 conformational changes in mammalian cells
Source: Cell Stress Chaperones. 2024 Oct 10;29(6):709–17. doi: 10.1016/j.cstres.2024.10.002 (PMC11532808; doi:10.1016/j.cstres.2024.10.002)
Supplement: Supplementary file 1 — Supplementary material [file mmc1.docx]

**Table S1 – DNA oligo sequences used in cloning.**

Blue – epitope tags; green – restriction enzyme sites; red – point mutations

| **Primer Name** | **Sequence** |
| --- | --- |
| BamHI-FLAG-mCherry-F | CTGTCGGATCCATGGATTACAAGGATGACGATGACAAAGGGAGTGAGCAAGGGCGAGGAGGA |
| mCherry-EcoRV-R | CTGTCGATATCCTTGTACAGCTCGTCCATGC |
| EcoRV-PP5-F | CTGTCGATATCGCGGAGGGCGAGAGGACTGA |
| PP5-XhoI-R | CTGTCCTCGAGCATCATTCCTAGCTGCAGCA |
| XhoI-GFP-F | CTGTCCTCGAGGTGAGCAAGGGCGAGGAGCT |
| GFP-His-XbaI-R | CTGTCTCTAGATCAATGATGGTGATGATGGTGCTTGTACAGCTCGTCCATGC |
| BamHI-FLAG-PP5-F | CTGTCGGATCCATGGATTACAAGGATGACGATGACAAGGGAGCGGAGGGCGAGAGGACTGA |
| PP5-His-XbaI-R | CTGTCTCTAGATCAATGATGGTGATGATGGTGCATCATTCCTAGCTGCAGCA |
| PP5-ΔαJ-XhoI-R | ATCAGCTCGAGTCAATAGGCCATGGGCTT |
| PP5-K430R-F | ATCCGCAGCCACGAAGTCCGTGCCGAGGGCTACGAGGTG |
| PP5-K430R-R | CACCTCGTAGCCCTCGGCACGGACTTCGTGGCAGCGGAT |
| PP5-K430A-F | ATCCGCAGCCACGAAGTCGCTGCCGAGGGCTACGAGGTG |
| PP5-K430A-R | CACCTCGTAGCCCTCGGCAGCGACTTCGTGGCAGCGGAT |
| PP5-K430Q-F | ATCCGCAGCCACGAAGTCCAAGCCGAGGGCTACGAGGTG |
| PP5-K430Q-R | CACCTCGTAGCCCTCGGCTTGGACTTCGTGGCAGCGGAT |
| PP5-K97E/R101E-F | GACAAGAAGTACATCGAGGGTTATTACGAGCGGGCTGCCAGCAAC |
| PP5-K97E/R101E-R | GTTGCTGGCAGCCCGCTCGTAATAACCCTCGATGTACTTCTTGTC |
| PP5-H304Q-F | TTCACCTCCTTCGAGGCAACCAAGAGACAGACAACATGAACCA |
| PP5-H304Q-R | TGGTTCATGTTGTCTGTCTCTTGGTTGCCTCGAAGGAGGTGAA |
| PP5-T362E-F | TGTTCAGTGAAGACGGTGTCGAACTGGATGACATCCGGAAAAT |
| PP5-T362E-R | ATTTTCCGGATGTCATCCAGTTCGACACCGTCTTCACTGAACA |
| PP5-delaJ-FRET-F | GTCAAGCCCATGGCCTATCTCGAGGTGAGCAAGGGC |
| PP5-delaJ-FRET-R | GCCCTTGCTCACCTCGAGATAGGCCATGGGCTTGAC |
